# Dietary n-3 PUFA Protects Mice from Con A Induced Liver Injury by Modulating Regulatory T Cells and PPAR-γ Expression

**DOI:** 10.1371/journal.pone.0132741

**Published:** 2015-07-15

**Authors:** Min Lian, Wenjing Luo, Yongheng Sui, Zhiping Li, Jing Hua

**Affiliations:** 1 Division of Gastroenterology and Hepatology, Ren Ji Hospital, School of Medicine, Shanghai Jiao Tong University, Shanghai Institute of Digestive Disease, Shanghai, P.R. China; 2 Department of Medicine, Johns Hopkins University, Baltimore, Maryland, United States of America; CIMA. University of Navarra, SPAIN

## Abstract

**Background:**

Dietary n-3 polyunsaturated fatty acids (PUFA) exert anti-inflammatory and immunoregulatory effects through down-regulating the innate and adoptive immune response. However, the effect of dietary n-3 PUFA on CD4^+^CD25^+^ regulatory T cells (Tregs) is unclear.

**Aims:**

The current study was to examine the relationship between n-3 PUFA and Tregs as well as their immunoregulatory effect in immune-mediated liver injury.

**Methods:**

The mice model feeding with n-3 PUFA-enriched diet was established and Tregs were analyzed. Effect of docosahexaenoic acid (DHA) on Tregs proliferation and induction was determined in vitro. The potential immunotherapeutic effect of dietary n-3 PUFA was investigated through Con A-induced hepatitis model.

**Results:**

Long-term administration of dietary n-3 PUFA significantly increased hepatic Tregs and modulated their phenotype. n-3 PUFA or DHA directly increased natural Tregs (nTreg) proliferation but didn’t increase inducible Tregs (iTreg). In addition, the expression of peroxisome proliferator activated receptor gamma (PPAR-γ), transforming growth factor β (TGF-β) and interleukin (IL)-10 were significantly up-regulated in n-3 PUFA-enriched diet-fed mice. Finally, n-3 PUFA-enriched diet alleviated liver injury induced by Con A and down-regulated pro-inflammatory cytokines expression, accompanied by increased PPAR-γ expression.

**Conclusion:**

Dietary n-3 PUFA enhanced Tregs generation through up-regulating PPAR-γ and TGF-β expression, and protected mice from Con A-induced liver injury. This finding provides a promising potential therapeutic method in treating inflammatory and autoimmune disease.

## Introduction

Dietary n-3 polyunsaturated fatty acids (n-3 PUFA) possess significant immunosuppressive and anti-inflammatory properties, they exert beneficial effects with respect to some inflammatory diseases and immune-mediated diseases [1–4]. Docosahexaenoic acid (DHA, 22:6, n-3) and eicosapentaenoic acid (EPA, 20:5, n-3) are major n-3 PUFAs in fish oil. As an endogenous nuclear receptor ligand, n-3 PUFA can up-regulate peroxisome proliferator-activated receptor gamma (PPAR-γ) expression in vivo and in vitro. PPAR-γ, a member of the nuclear receptors and a ligand-dependent transcription factor, can regulate insulin and glucose metabolism, and also has the ability to down-regulate the immune response of cells in the innate and adaptive immune system [5,6]. Therefore, the underlying anti-inflammatory mechanism of n-3 PUFA may be dependent on PPAR-γ.

CD4^+^CD25^+^ regulatory T cells (Tregs) play a critical role in maintaining peripheral self-tolerance and controlling pathogenic-specific responses. There are several subtypes of Tregs as described: thymus-generated natural Tregs (nTregs) as well as peripheral antigen induced CD4^+^ CD25^+^ Foxp3^+^ Treg (iTreg), interleukin (IL)-10-(Tr1) and transforming growth factor β (TGF-β)-producing (Th3) Treg [7,8]. Considerable evidence has been accumulated for the suppressive and immunomodulatory effects of Tregs on different conditions of liver disease, such as hepatocellular carcinoma, autoimmune hepatitis and liver transplant [9–11]. The frequency and functional deficiency of Tregs plays an important role in the pathogenesis of autoimmune liver disease [12].

Earlier studies on the anti-inflammatory and immunoregulatory effect of n-3 PUFA have focused on its ability to down-regulate the immune response including inhibiting activated T cell proliferation, decreasing cytokines production and regulating the antigen presentation cells (APCs) function [13,14]. Recently, investigations have focused on the relationship between n-3 PUFA and Tregs. However, results were complex and sometimes contradictory. Some studies demonstrated that EPA could induce Tregs production and increase PPAR-γ expression, and even prolonged survival of cardiac allografts in EPA-treated mice [15,16]. However, others found DHA reduced suppressive and migratory functions of Tregs [17].

Effect of dietary n-3 PUFA in immune-mediated liver injury was not well studied. In the current study, we established long-term administration of n-3 PUFA-enriched diet mice model and in vitro DHA treatment experiment to characterize the relationship between dietary n-3 PUFA and Tregs. Furthermore, the potential immunotherapeutic effect of dietary n-3 PUFA in immune-mediated liver injury was investigated through Con A-induced hepatitis model.

## Materials and Methods

### Animal experiments

Adult (age 6–8 week) male wild type C57BL/6 mice were purchased from Shanghai SLAC Laboratory Animal Co. Ltd. The mice were fed with commercial diets (Medicience Ltd, China) containing either different concentrations of n-3 PUFA-enriched diet (20% or 45% kcal from fat provided by fish oil-Menhaden Oil respectively, Table 1) or normal amounts of fat diet (ND, 15% kcal from fat) for 4–12 weeks. The low concentration of n-3 PUFA-enriched diet (L-PUFA) contained 4.62% of DHA and 2.8% of EPA, and the high concentration of n-3 PUFA-enriched diet (H-PUFA) contained 12.4% of DHA and 7.35% of EPA. All mice were housed under specific-pathogen free conditions in the animal facility of Ren Ji Hospital. The mice were sacrificed by injecting with 4% chloral hydrate (100μl/10g) intraperitoneally after feeding different diets for 4–12 weeks. All animal experiments fulfilled Shanghai Jiao Tong University criteria for the humane treatment of laboratory animals and were approved by the Ren Ji Hospital Animal Care and Use Committee (SYXK 2011–0121).

**Table 1:** Diet composition.

	ND		L-PUFA		H-PUFA	
%	kcal		kcal		kcal	
Protein	25		25		25	
Carbohydrate	60		55		30	
Fat	15		20		45	
Total	100		100		100	
Ingredient	g	kcal	g	kcal	g	kcal
Casein	250	1000	250	1000	250	1000
L-Cystine	3.5	14	3.5	14	3.5	14
Corn Starch	126	504	115	461	62	248
Maltodextrin	173	692	159	634	85	341
Sucrose	299	1196	274	1096	147	588
Cellulose	50	0	50	0	50	0
Soybean oil	25	225	0	0	0	0
Menhaden Oil	0	0	90	811	203	1825
Lard	42.5	383	0	0	0	0
Mineral Mix	45	0	45	0	45	0
Vitamin Mix	10	40	10	40	10	40
Choline Bitartrate	2	0	2	0	2	0
Dye	0.05	0	0.05	0	0.05	0
Total	1026.05	4054	998.55	4056	857.55	4056

For immune-mediated liver injury experiments, mice were fed with either L-PUFA diet or ND diet for 6–8 weeks, and then were injected with a single dose of Concanavalin A (Con A, 10mg/kg, Sigma-Aldrich Corporation, St. Louis, MO) via tail vein. The mice were sacrificed 24 hours later, and the serum and liver and spleen tissue were collected.

### Hepatic mononuclear cells and splenocytes isolation and labeling

Hepatic mononuclear cells (HMNCs) and splenocytes were isolated as previously described [18]. Single-cell suspensions of HMNCs and splenocytes were labelled with fluorescent conjugated antibodies against mouse CD4-FITC, CD25-PE (eBioscience), CD62L-PE Cy7, CTLA-4-PE (BD Pharmingen), as well as CD103-PerCP-Cy5.5 (Biolegend). Nonspecific binding was blocked with staining buffer supplemented with 2% mouse serum and anti-CD16/anti-CD32. For intracellular staining of Foxp3, mouse regulatory T cell staining Kit (eBioscience) was used. Cells were labeled with surface antibody, fixed and permeabilized with freshly prepared Fixation/Permeabilization working solution according to the manufacturer’s instructions. After permeabilization, cells were further labeled with anti-mouse foxp3 (clone FJK-16s) (eBioscience). After incubation and resuspension, HMNCs and splenocytes were evaluated by flow cytometry, and the data were analyzed using Cell Quest software (Becton Dickinson).

### In vivo Tregs proliferation assay

To assess the long-term proliferation of Tregs in steady state, mice feeding with either ND diet or L-PUFA diet for 4–12 weeks were administered 5-bromo-2-deoxyuridine (BrdU, 0.8mg/ml, Sigma) in drinking water for 7 consecutive days before sacrificed. Incorporation of BrdU was determined using a BrdU Flow kit (BD Pharmingen) according to the manufacturer’s instructions. Briefly, HMNCs and splenocytes were prepared as above. Cells were stained with surface antibody CD4 and CD25, then fixed and permeabilized. Finally, cells were stained with intracellular marker anti-BrdU and anti-Foxp3, and analyzed by four-color FACSCalibur (BD, New Jersey, USA).

### In vitro Tregs proliferation and induction assays

Single splenocyte suspensions were prepared from wild type mice. CD4^+^CD25^+^ Tregs and CD4^+^CD25^-^ T cells were purified using the Treg cell isolation kit (MACS, Miltenyi Biotec). The purity of the cell separation was 90–95%, as assessed by flow cytometry. Culture medium containing DHA was freshly made. DHA was dissolved in the pure ethanol to form the stock solution. The cell culture medium containing DHA was made by dissolving the DHA stock solution to RPMI solution with 1% bovine serum albumin (BSA).

To determine the direct effect of DHA on CD4^+^CD25^+^ Tregs proliferation, Tregs (5x10^4^ per well) were activated with anti-CD3/CD28 beads (0.1 beads/cell, Dynabeads, Invitrogen) and IL-2 (100U/ml) in the presence or absence of varying dose of DHA (6.25–50μM, Sigma) in RPMI medium containing 1% BSA for 3 days. Cells proliferation was assessed by incorporation of [^3^H] thymidine (0.5μCi/well), which was added for the last 16 h of culture.

To determine the effect of DHA on induction of Tregs from CD4^+^CD25^-^ Teff, Teffs were incubated (1x10^5^/well) in flat-bottom 96-well plate coated with anti-CD3 Ab (5μg/ml) in the presence of soluble anti-CD28 Ab (2μg/ml) with or without recombinant human TGF-β1 (2μg/ml, R&D Systems) and DHA (25μM, Sigma-Aldrich Corporation). Cells were cultured in RPMI 1640 medium supplemented with 2mM L-glutamine, 100U/ml penicillin, 100μg/ml streptomycin and 1% BSA for 3 days. At the end of 3 days, cells were harvested and stained with surface markers and intracellular Foxp3.

### RNA isolation and evaluation of cytokine gene expression

Total RNA was extracted from liver tissue using TRIzol reagent (Invitrogen, USA) and cDNA was synthesized from 2 μg of total RNA using Primescript RT Reagent kit (TaKaRa, Japan). For real-time PCR, 10 ng of template was used in a 10-μl reaction containing each primer, SYBR Green PCR Master Mix (TaKaRa, Japan). All reactions were performed in triplicate using the following cycling conditions: 30 s at 95°C, followed by 40 cycles of 95°C for 5 s, 60°C for 30 s and 72°C for 30 s using ABI Prism 7300 system (Applied Biosystems, USA). The mean value of the triplicate for each sample was calculated and expressed as cycle threshold (CT). The amount of gene expression was then calculated as the difference (ΔCT) between the CT value of the sample for the target gene and the mean CT value of the endogenous control (β-actin). The relative level of expression was measured as 2-ΔΔCT. Mouse primers (provided by Sangon Biotech Co., Shanghai, China) were as follows: IL-10: GTTACTTGGGTTGCCAAG and TTGATCATCATGTATGCTTC;

TGF-β: AGGGCTACCATGCCAACTTC and CCACGTAGTAGACGATGGGC;

IL-1β: TGCTGGTGTGTGACGTTCCC and TGAGGCCCAAGGCCACAGGTA;

TNF-α: TCTTCTCATTCCTGCTTGTGG and GGTCTGGGCCATAGAACTGA;

IL-6: GTTCTCTGGGAAATCGTGGA and GGAAATTGGGGTAGGAAGGA;

IFN-γ: AGAAATAGTTGAGGAGACAGAAAT and TTAGATGCATCAACCAAAGAAGTA;

PPAR-γ: GCCCTTTACCACAGTTGATTTCT and GTGATTTGTCCGTTGTCTTTCCT; PPAR-α: CGCGTGTGATAAAGCCATTG and CACGATGCTGTCCTCCTTGA;

β-actin: TGTTACCAACTGGGACGACA and CTGGGTCATCTTTTCACGGT.

### Western Blot analysis

Whole protein extracts of liver tissue were resolved by 8% SDS-PAGE (sodium dodecyl sulfate-polyacrylamide gel electrophoresis) according to standard procedures. The samples were then transferred to 0.45 um nitrocellulose membranes (Bio-Rad, Hercules, CA) and incubated overnight with antibodies to PPAR-γ, PPAR-α or the endogenous control GAPDH (Cell Signaling Technology, Danvers, MA, USA). Chemiluminescent signals were quantitated by density measurements with ImageJ software (NIH).

### Liver histology and serum alanine aminotransferase determination

Liver tissues were obtained when the mice were sacrificed. The right lobes of liver tissue from different groups of mice were fixed in 4% formaldehyde, and were prepared for hematoxylin and eosin (H&E) staining following routine methods. Serum ALT was determined by the spectrophotometric method as previously described [19].

### Statistical analysis

All continuous variables are expressed as mean ± standard deviation (SD). Statistical differences were determined by a student t test or one-way analysis of variance test. All analyses were two-tailed and performed using GraphPad Prism (GraphPad Software, San Diego, USA), *p* value <0.05 was considered statistically significant.

## Results

### n-3 PUFA-enriched diet increases hepatic Tregs

**Fig 1:**
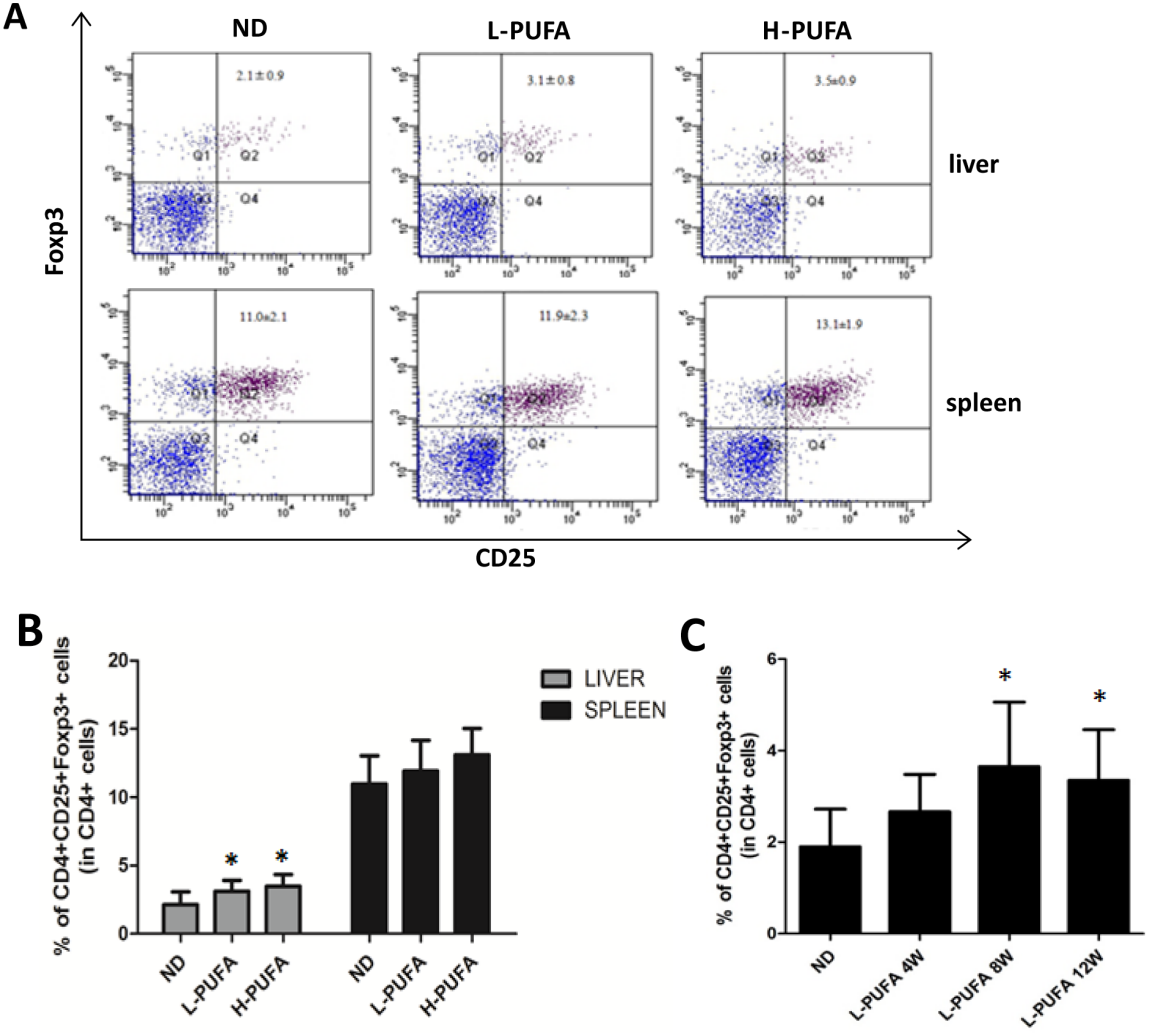
n-3 PUFA-enriched diet increases hepatic Tregs. Hepatic mononuclear cells (HMNCs) and splenocytes were isolated from mice fed with normal diet (ND) and different concentrations of n-3 PUFA-enriched diet (L-PUFA and H-PUFA) for 12 weeks. Tregs were identified by CD4, CD25, and Foxp3 fluorescent staining (n = 10 each group). A) Representative dot plots of CD25^+^Foxp3^+^Tregs in the right upper quadrant (gated from CD4^+^ cells). B) Mean (±SD) percentage of hepatic and splenic Tregs among CD4^+^ cells from ND, L-PUFA and H-PUFA fed mice. C) Mean (±SD) percentage of hepatic Tregs among CD4^+^ cells from mice fed with n-3 PUFA-enriched diet for different periods (n = 5 each group). *p<0.05 vs ND mice

**Fig 2:**
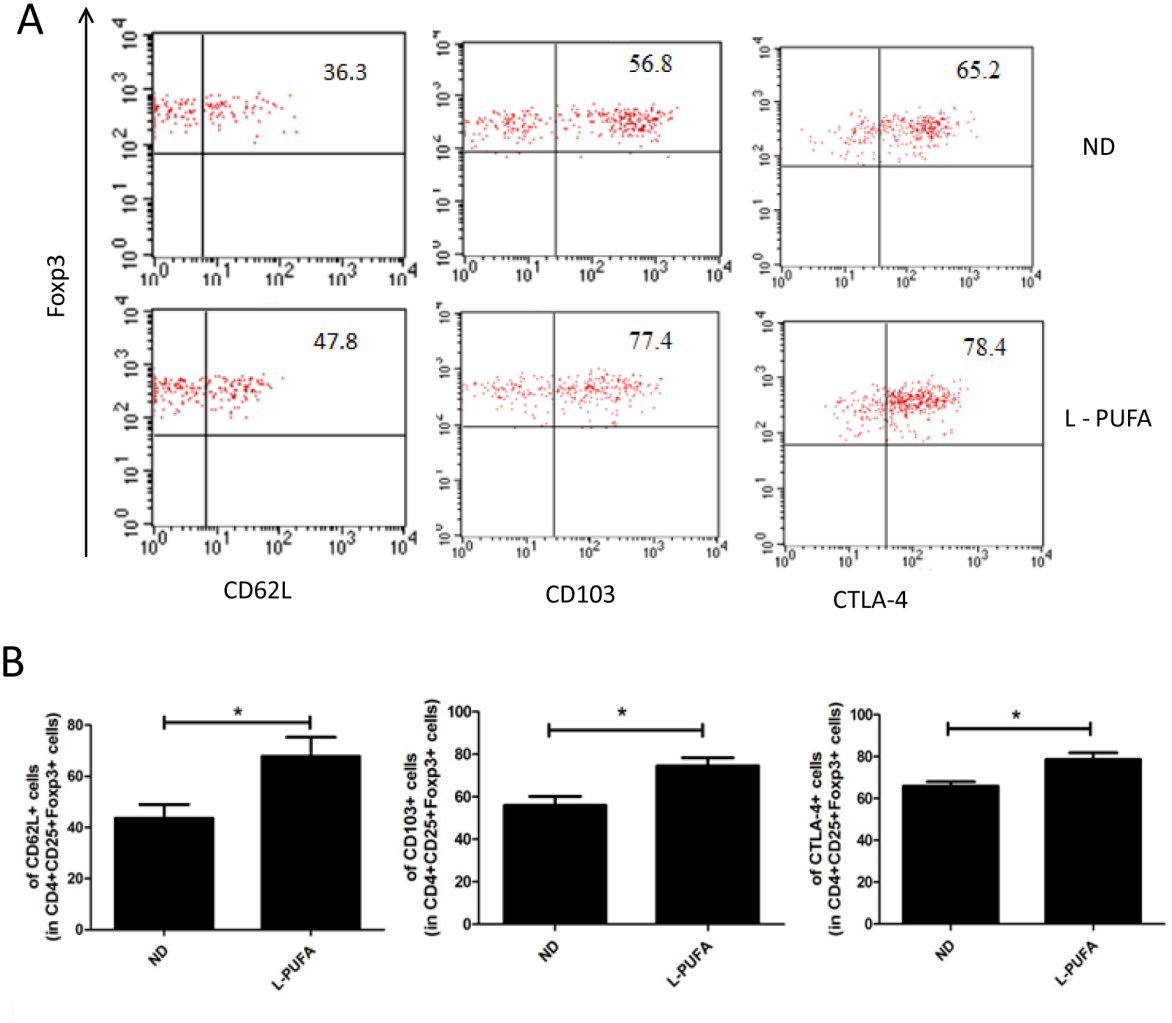
The phenotype of hepatic Tregs. HMNCs were isolated from mice consuming normal diet (ND) or low concentration of n-3 PUFA-enriched diet (L-PUFA). The expression of CD62L, CD103 and CTLA-4 on hepatic Tregs (CD4^+^CD25^+^Foxp3^+^) was analyzed by flow cytometry. A) Representative dot plots of CD62L, CD103 and CTLA-4 expression on Tregs in the right upper quadrant. B) Mean (±SD) percentage of CD62L, CD103 and CTLA-4 expression among Tregs (n = 5 each group). *p<0.05

Dietary n-3 PUFA has already been used in many chronic inflammatory conditions and autoimmune diseases due to its anti-inflammatory property [3,4]. Few studies have investigated the relationship between n-3 PUFA and Tregs. Here, we set up a mice model feeding with diet rich with n-3 PUFA to determine its effect on Tregs. After 12 weeks feeding, the percentage of hepatic CD25^+^Foxp3^+^ Tregs among CD4^+^ T cells was significantly higher in low and high concentration of n-3 PUFA-enriched diet-fed mice than that in ND diet group (L-PUFA vs ND: 3.12±0.80 vs. 2.15±0.92, *p*<0.05; H-PUFA vs ND:3.48±0.86 vs. 2.15±0.92, *p*<0.05, Fig 1A and 1B). There was no significant difference between the high and low concentration of n-3 PUFA-enriched diet groups. Although there was increased calorie intake from fat in both n-3 PUFA-enriched diet groups, the body weights were not statistically different from those of ND diet group. Furthermore, there was no hepatic steatosis and inflammation on liver histology in both n-3 PUFA-enriched diet groups (S1 Fig). The total HMNCs and CD4^+^ T cells were similar in these groups, and the numbers of Kupffer cell were similar in liver histology (S1 Fig). However, n-3 PUFA-enriched diet-induced Tregs expansion was liver specific, no significant change of Tregs was observed in spleen (Fig 1A and 1B). Moreover, the dynamic analysis showed that long-term administration of n-3 PUFA-enriched diet induced significant hepatic Tregs expansion (Fig 1C). We also investigated some major markers reported to be characteristics of Tregs associated with their homing and suppression function in L-PUFA diet-fed mice. CD62L, CD103 and CTLA-4 of Tregs were more highly expressed in L-PUFA diet-fed mice than in ND diet-fed mice (Fig 2A and 2B). Together, these results showed that long-term administration of dietary n-3 PUFA induced generation of hepatic Tregs, as well as changed their phenotype.

### Effect of n-3 PUFA on Tregs proliferation in vivo

**Fig 3:**
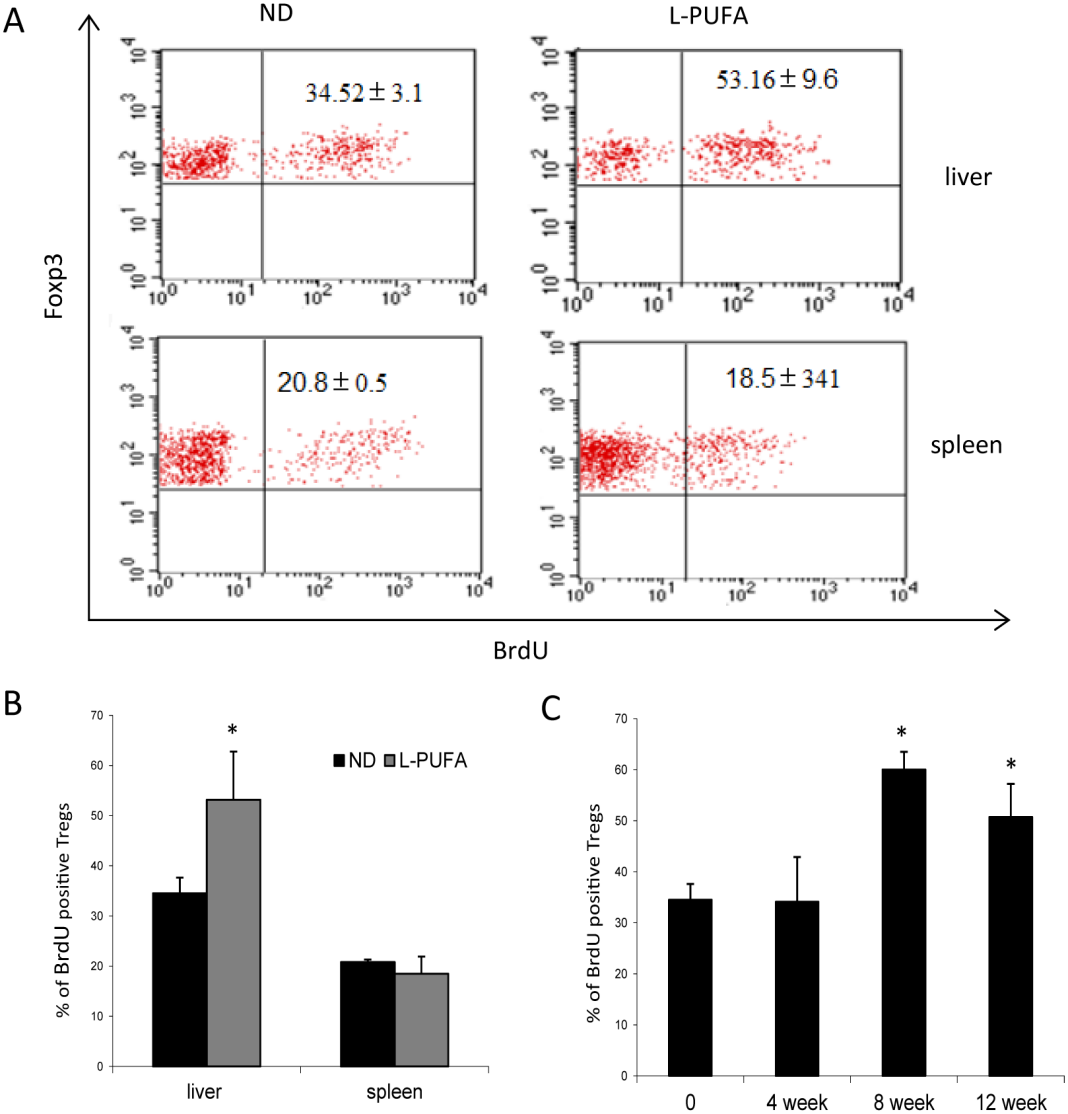
Effect of n-3 PUFA on the proliferation of Tregs in vivo. To assess the proliferation of Tregs in vivo, mice consuming ND diet or low concentration of n-3 PUFA (L-PUFA) diet for 4–12 weeks were administered BrdU (0.8mg/ml) in drinking water for 7 consecutive days before sacrifice. HMNCs and splenocytes were isolated as above and fluorescent stained. A) Representative dot plots of BrdU expression in Foxp3^+^ cells in the right upper quadrant (gated from CD4^+^CD25^+^ cells). B) Mean (±SD) percentage of BrdU^+^ Tregs in liver and spleen (n = 5 each group). C) Mean (±SD) percentage of hepatic BrdU^+^ Tregs from mice fed with L-PUFA-enriched diet for different periods (n = 5 each group). *p<0.05 vs ND mice

Since n-3 PUFA-enriched diet increased hepatic Tregs, we assessed whether n-3 PUFA had direct effect on the long-term turnover or proliferation of Tregs. We used L-PUFA diet-fed mice as the representative due to the similar increase of hepatic Tregs in both groups. Mice feeding with either ND diet or L-PUFA diet were administered BrdU in drinking water for 7 consecutive days before sacrifice. Incorporation of BrdU in Tregs indicated cells proliferation. As shown in Fig 3A and 3B, the percentage of hepatic BrdU^+^Foxp3^+^ Tregs (gate from CD4^+^CD25^+^) in L-PUFA diet-fed mice was significantly higher than that in ND diet mice. However, there was no significant difference on spleen Tregs proliferation between two groups. The result of time-course analysis showed that hepatic Tregs enhanced proliferation after long time administration of n-3 PUFA-enriched diet (Fig 3C), which was consistent with the result of hepatic Tregs expansion. These results indicated that n-3 PUFA-enriched diet increased hepatic Tregs through promoting their proliferation.

### Effect of n-3 PUFA on Tregs proliferation and induction in vitro

**Fig 4:**
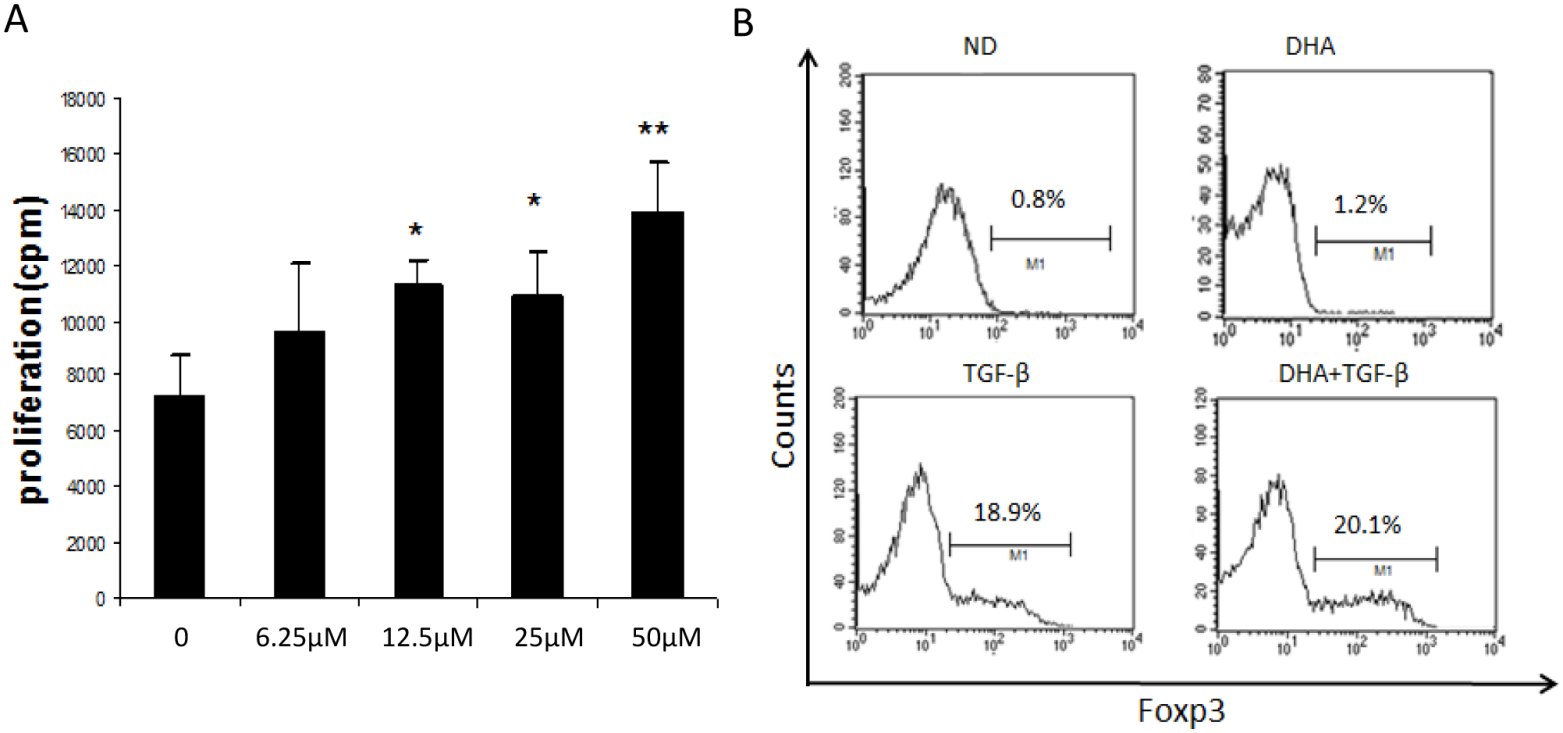
Effect of DHA on Tregs proliferation and induction in vitro. CD4^+^CD25^+^ (Tregs) and CD4^+^CD25^-^ (Teffs) were isolated from spleen of wild type mice by MACS. A) Effect of DHA on nTregs proliferation. Purified CD4^+^CD25^+^ Tregs (5x10^4^/well) were activated with anti-CD3/CD28 beads and IL-2 (100U/ml) in the presence or absence of varying dose of DHA (6.25–50μM) for 3d. Mean (±SD) results were graphed, *p<0.05,**p<0.01. B) Effect of DHA on Tregs induction. Teffs were incubated (1x10^5^/well) in flat-bottom 96-well plate coated with anti-CD3 Ab (5μg/ml) in the presence of soluble anti-CD28 Ab (2μg/ml) with or without TGF-β1 (2μg/ml) and DHA (25μM). Representative histogram of Foxp3^+^cells (gated from CD4^+^CD25^+^cells) was shown.

Next, we’d like to determine whether DHA, a major n-3 PUFA, could directly increase Tregs proliferation in vitro. Purified CD4^+^CD25^+^ Tregs by MACS were stimulated with anti-CD3/CD28 beads in the presence of IL-2 and various doses of DHA. As expected, DHA significantly enhanced CD4^+^CD25^+^ Tregs proliferation in a dose-dependent manner (Fig 4A).

Because Tregs could be induced from naïve CD4^+^ T cells in the presence of exogenous TGF-β under the activated status, next we’d like to clarify whether DHA could affect the generation of inducible Tregs (iTregs) in vitro. Consistent with previous reports [20], CD4^+^CD25^-^ Teff cells could be converted to CD4^+^CD25^+^Foxp3^+^ Tregs when they were activated by polyclonal stimulation in the presence of exogenous TGF-β (Fig 4B). However, the addition of DHA had no further enhanced effect on TGF-β mediated induction of iTregs, and DHA alone failed to induce iTregs. Taken together, n-3 PUFA increased nTregs proliferation but didn’t induce iTregs.

### n-3 PUFA-enriched diet modulates the expression of PPAR-γ, TGF-β and IL-10

**Fig 5:**
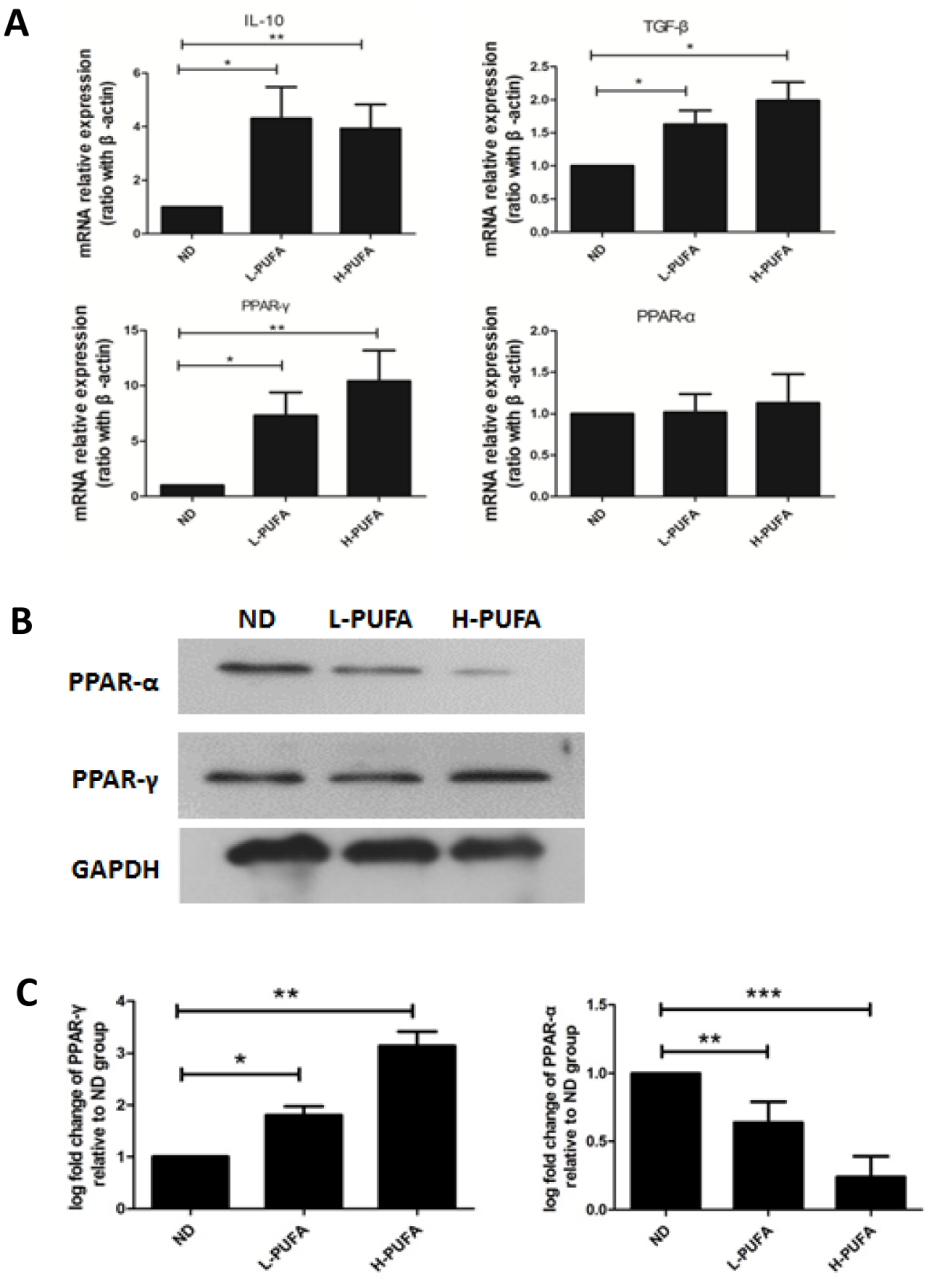
Effects of n-3 PUFA-enriched diet on hepatic anti-inflammatory cytokines and nuclear receptors expression. Wild-type C57BL/6 mice were fed with ND, L-PUFA and H-PUFA diet for 12 weeks. A) Hepatic IL-10, TGF-β, PPAR-γ and PPAR-α mRNA expressions. B) PPAR-γ and PPAR-α protein expressions measured by Western blot. C) Mean (±SD) protein expression of PPAR-γ and PPAR-α. *p<0.05,**p<0.01, ***p<0.001.

Our in vivo and in vitro studies have shown that n-3 PUFA-enriched diet or DHA directly increased Tregs proliferation. Next, we’d like to explore the candidate factors which may contribute to Tregs generation. Recent studies have shown that activation of PPAR-γ induced CD4^+^ T-cells anergy and loss of PPAR-γ in T cells impaired Tregs [21]. Moreover, EPA increased Tregs but a PPAR-γ antagonist abrogated the increase of Tregs. These results suggested that PPAR-γ contributes to generation of Tregs. Indeed, we found PPAR-γ expression in liver tissues was significantly elevated at mRNA level and protein level in both low and high concentration of n-3 PUFA group (Fig 5A, 5B and 5C). In addition, previous studies showed that cytokine IL-10 and TGF-β were involved in the generation and maintenance of Tregs [22–25]. In the current study, we also found IL-10 and TGF-β expression were significantly higher in liver tissues from n-3 PUFA-enriched diet-fed mice than those of ND diet group (Fig 5A).

### n-3 PUFA-enriched diet protects mice from Con A-induced liver injury

**Fig 6:**
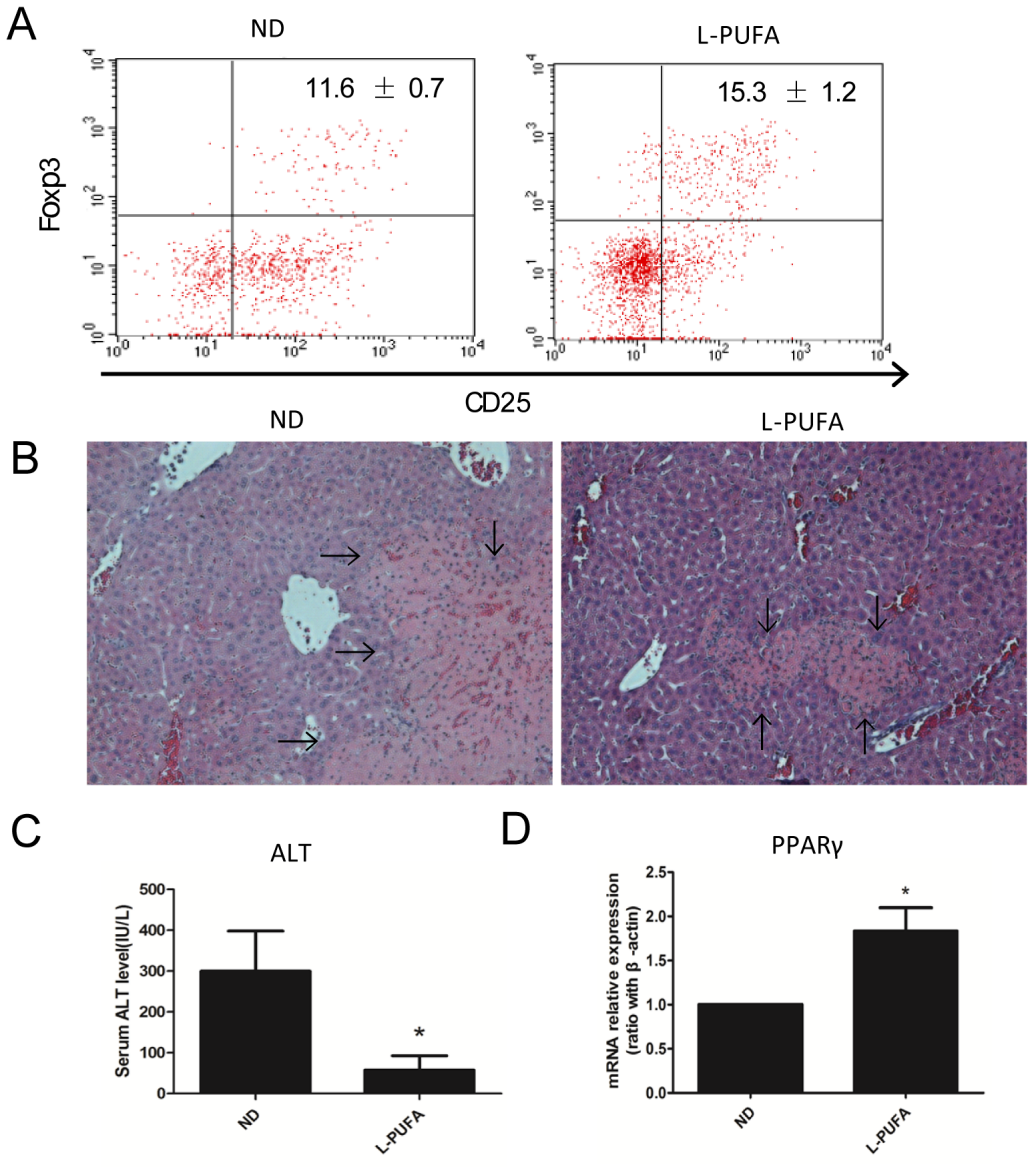
n-3 PUFA-enriched diet protects mice from Con A-induced liver injury. Wild-type mice were injected with Con A after consuming ND diet or L-PUFA diet for 6 or 8 weeks. HMNCs were isolated and Tregs were identified as above, and the serum and liver tissue were obtained 24 hours after Con A injection. A) Representative dot plots of CD25^+^Foxp3^+^Tregs (gated from CD4^+^ cells). B) Representative liver histology using H&E staining, the arrows indicated damaged areas including necrosis. C) Mean (±SD) of serum ALT levels. D) Hepatic PPAR-γ mRNA expressions. (n = 5 each group), *p<0.05.

**Fig 7:**
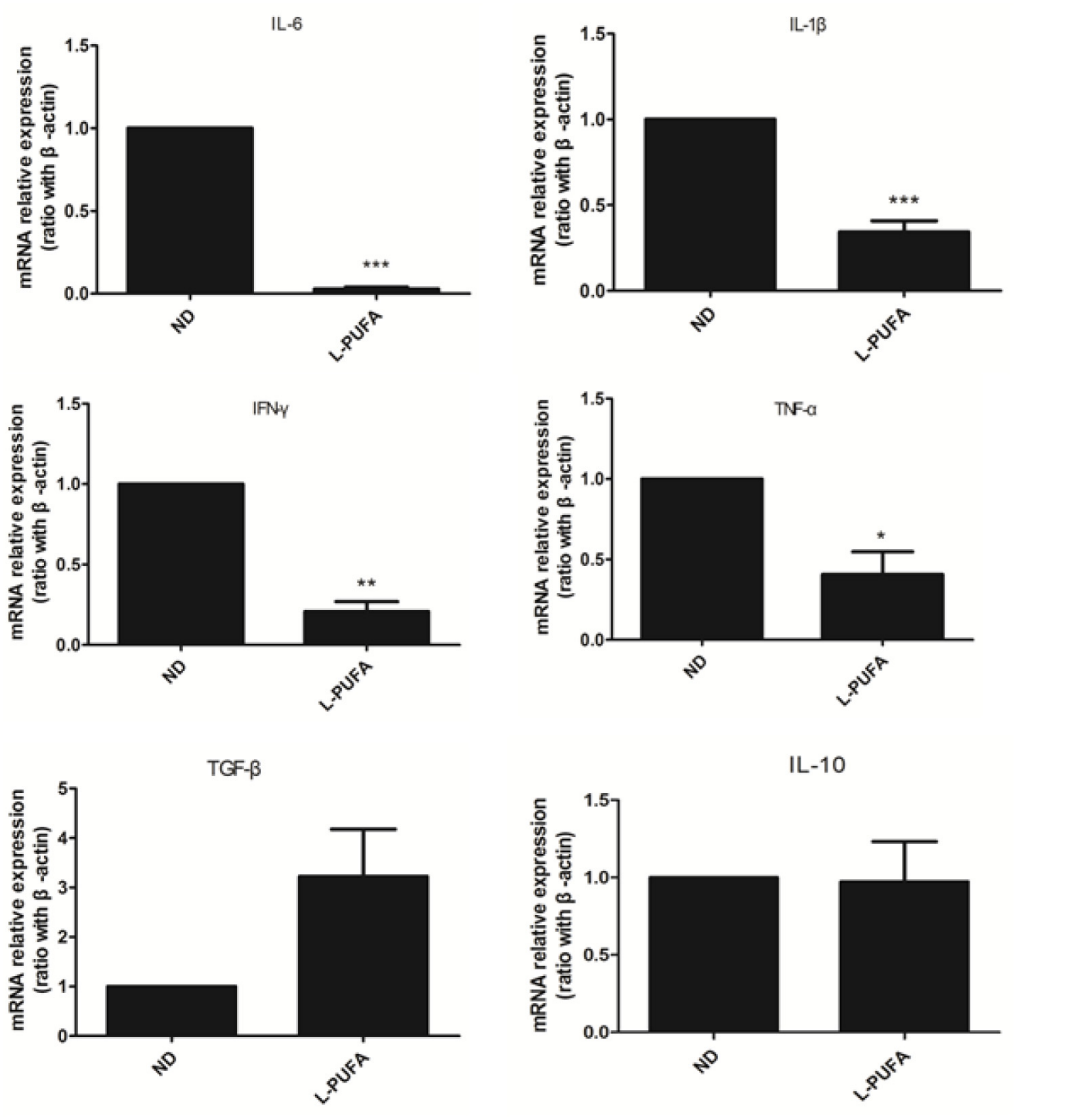
Hepatic inflammatory-related cytokines expression in Con A-induced liver injury model. Pro-inflammatory and anti-inflammatory cytokines mRNA expressions in liver tissues from ND diet or L-PUFA diet feeding mice, (n = 5 each group), *p<0.05,**p<0.01,***p<0.001.

Tregs play a critical role in maintaining peripheral self-tolerance and controlling inflammation. To evaluate whether dietary n-3 PUFA-mediated Tregs expansion has any biological relevance, mice were injected with Con A to elicit hepatitis, which was mainly mediated by activated T cells. Consistent with the previous report [9], Con A treatment increased hepatic Tregs, and mice consuming n-3 PUFA-enriched diet had more hepatic Tregs than those consuming ND diet (PUFA vs ND: 15.3±1.2 vs 11.6±0.7, *p*<0.05, Fig 6A). As expected, mice consuming n-3 PUFA-enriched diet had less liver injury as reflected by liver histology analysis (Fig 6B) and serum ALT levels (Fig 6C) than those with ND diet. In addition, PPAR-γ expression in liver in n-3 PUFA-enriched diet-fed mice was significantly increased (Fig 6D). Moreover, the pro-inflammatory cytokines expression (IL-6, IL-1β, IFN-γ and TNF-α) were significantly reduced in n-3 PUFA-enriched diet-fed mice (Fig 7). TGF-β gene expression increased, although no significant change was observed (Fig 7). These results demonstrated that n-3 PUFA-enriched diet may protect mice from Con A-induced liver injury through Tregs expansion and up-regulation of PPAR-γ expression, and subsequently down-regulation of pro-inflammatory cytokines expression.

## Discussion

Long chain n-3 PUFAs, such as DHA and EPA, have been shown to exert significant immunosuppressive and anti-inflammatory effects both in rodents [26] and in humans [27]. These beneficial effects were attributed to their ability to down-regulate the immune response. Our current study obtained several new evidence to support the theory that Tregs were one of the key components of n-3 PUFA induced anti-inflammatory and immunomodulatory effects: (a) Long-term administration of n-3 PUFA-enriched diet increased hepatic Tregs. (b) In vivo and in vitro studies showed n-3 PUFA increased nTregs proliferation but didn’t induce iTreg. (c) n-3 PUFA-enriched diet protected mice against Con A induced liver injury through Tregs expansion as well as increased PPAR-γ expression and subsequently down-regulation of inflammatory cytokines expression.

There is accumulating evidence that dietary n-3 PUFA has beneficial effects in many chronic inflammatory conditions and autoimmune diseases due to its anti-inflammatory property [28,29]. However, these studies focused on the ability of n-3 PUFA to down-regulate the immune response. In the current study, we established a long-term administration of n-3 PUFA-enriched diet mice model to characterize the relationship between n-3 PUFA and Tregs. We found that administration of n-3 PUFA-enriched diet increased hepatic Tregs generation. And in vivo and in vitro experiments demonstrated that n-3 PUFA-mediated Tregs expansion depended on nTregs proliferation. In addition, some Tregs marker molecules including CD103 [30], CTLA-4 and CD62L involved in Tregs suppressive role were expressed at higher levels in n-3 PUFA-fed mice than those in ND diet-fed mice. The elevated expression of these important molecules indicated Tregs phenotype change and subsequent enhanced suppressive function. Our results were consistent with previous report on the relationship between n-3 PUFA and Tregs. Iwami found EPA-treated mice demonstrated increased Foxp3^+^ cells in transplanted hearts and prolonged survival [16]. However, unlike our long-term administration of n-3 PUFA, very short-term (only one injection) but high dose of EPA was used in their experiment. To obtain a more physiologic context, in the current study we set both low and high concentration of n-3 PUFA-enriched diet-fed mice models, in which caloric provided by fat was 20% and 45% respectively. The results showed that increase of Tregs in the low concentration of n-3 PUFA-enriched diet group was similar to that in the high concentration group. Therefore, the low concentration of n-3 PUFA-enriched diet, in which the calorie was more close to the physiologic level, is probably more feasible and recommended in clinical practice.

Dietary n-3 PUFA presented in fish oil, and their metabolites, provide the ligands for nuclear receptors. As PPAR-γ natural ligands, n-3 PUFA could up-regulate PPAR-γ expression in macrophages and reduce an inflammatory response [31–33]. In the current study, we found PPAR-γ expression in liver tissues was significantly up-regulated both in low concentration and high concentration of n-3 PUFA-enriched diet mice model. The other nuclear receptor PPAR-α expression was decreased at protein level, which was associated with lipid metabolism and oxidative stress. Wohlfert EA et al found that ciglitazone, a synthetic PPAR-γ ligand, exerted the immunoregulatory effect in a model of graft-vs-host disease dependent on the presence of nTregs that expressed PPAR-γ [34]. Iwaim D et al reported that administration of antagonist of PPAR-γ abolished EPA-induced prolonged survival of cardiac allografts and generation of Tregs [16]. Zhao W et al demonstrated that PPAR-γ agonists pioglitazone significantly increased proliferation and function of lupus Tregs [35]. These results indicated that both natural and synthetic PPAR-γ ligand could increase Tregs frequency and function through PPAR-γ-dependent mechanism. Recently, an elegant study conducted by Cipolletta.D et al indicated PPAR-γ was a critical molecular orchestrator on accumulation of visceral adipose tissue (VAT) Tregs using Treg-Pparγ transgenic mice [36]. Taken together, we speculated that up-regulation of PPAR-γ expression after long-term administration of n-3 PUFA-enriched diet may contribute to the increase of hepatic Tregs.

IL-10 and TGF-β are major immune suppressive cytokines which are required for the function of Tregs [23,24]. In addition, these cytokines are also involved in the generation and maintenance of Tregs. Previous studies have shown TGF-β regulated the expression of Foxp3 and expanded Foxp3-expressing CD4^+^CD25^+^ T cells in vivo [37]. Recently, Han et al found administration of fermented fish oil increased the expression of TGF-β and Foxp3 at the sites of inflammation, which was associated with enhancement of Treg population [38]. In addition, EPA could up-regulate the expression of anti-inflammatory cytokines (IL-10) and increase the number of Foxp3^+^ Tregs [39]. In our study, we found TGF-β and IL-10 expression in liver tissues were increased after feeding n-3 PUFA-enriched diet, which may also contribute to the generation of hepatic Tregs, at least partly.

Tregs play a pivotal role in regulating and controlling immune response through cell-cell contact or the production of regulatory cytokines such as IL-10 and TGF-β [7]. Given the ability of n-3 PUFA-enriched diet to enhance hepatic Tregs, we wondered whether these diets when fed to mice could protect against Con A induced liver injury. As expected, mice consuming an n-3 PUFA-enriched diet had less liver injury and more hepatic Tregs than those consuming the ND diet. Con A-induced liver injury was a widely accepted model of immune-mediated liver disease which was characterized with pronounced activated T cells and significant production of pro-inflammatory cytokines [9]. In the current study, we found pro-inflammatory cytokines expressions were significantly reduced in n-3 PUFA-enriched diet-fed mice. We speculated that enhanced PPAR-γ expression and associated increase of Tregs generation were the underlying mechanism of n-3 PUFA-enriched diet in the immune-mediated liver injury. Recently, Cipolletta.D et al found that PPAR-γ could cooperate with Foxp3 to impose a Treg phenotype on naïve CD4^+^ T cells in vitro [36]. Therefore, up-regulation of PPAR-γ expression enhanced Tregs frequency and their function. However, other possible mechanism of anti-inflammatory effect of n-3 PUFA may also be involved. Recently, molecular investigation has shown that the anti-inflammatory response exerted by PPAR-γ was partly through its interactions with some transcription factors, such as AP1 and NF-κB, thereby interfering with their DNA binding and subsequently down-regulating the expression of the target cytokines [40]. Therefore, n-3 PUFA-mediated PPAR-γ activation maybe the key step to implement their anti-inflammatory and anti-autoimmune responses.

In conclusion, our results indicated that dietary n-3 PUFA could generate Tregs and modulate their function. Dietary n-3 PUFA protected mice from Con A-induced liver injury through Tregs expansion as well as increased PPAR-γ expression and subsequently down-regulation of pro-inflammatory cytokines expression. This finding provided a promising potential therapeutic method in treating inflammatory and autoimmune disease.

## Supporting Information

S1 Fig The body weight, liver histology and Kupffer cells of mice fed with n-3 PUFA-enriched diet. Wild-type C57BL6 mice were fed either with normal diet (ND) or different concentrations of n-3 PUFA-enriched diet (L-PUFA and H-PUFA) for 12 weeks. A) animal weight (n = 10/group). There was no significant difference of the body weights among the three groups. B) Serum ALT level, C) liver histology (upper) and immunohistochemistry (IHC, lower). Kupffer cells were indicated by detection of the F4/80 positive expression of brown yellow staining in cytoplasm (200x magnification), the arrows indicated Kupffer cells. D) Mean (±SD) number of Kupffer cells, ten fields per section from each mouse were analyzed (n = 4/group).
(TIF)
