# Supplementary figures and images for: Dietary n-3 PUFA Protects Mice from Con A Induced Liver Injury by Modulating Regulatory T Cells and PPAR-γ Expression
Source: PLoS One. 2015 Jul 15;10(7):e0132741. doi: 10.1371/journal.pone.0132741 (PMC4503783; doi:10.1371/journal.pone.0132741)

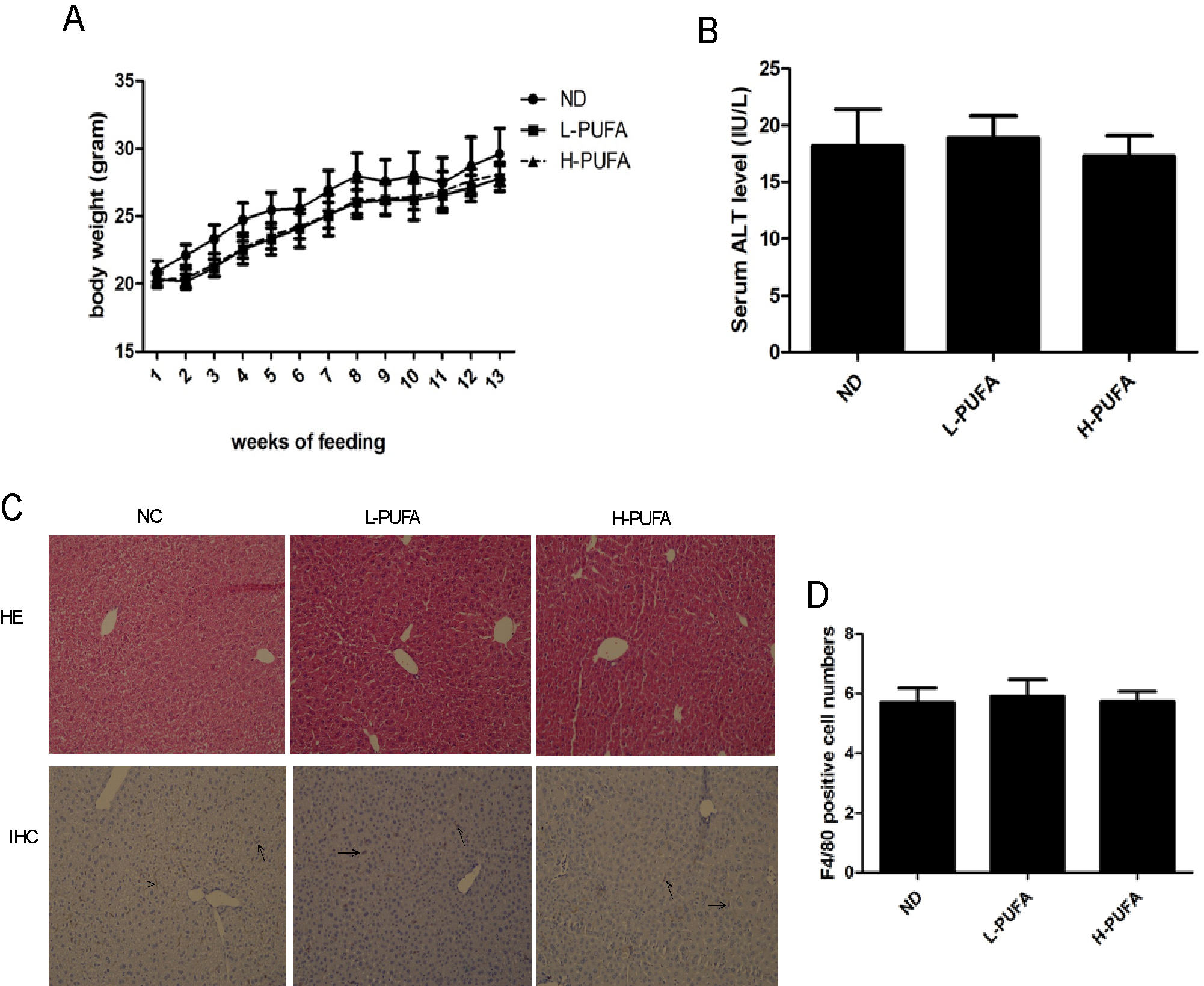

Supplement: S1 Fig — Wild-type C57BL6 mice were fed either with normal diet (ND) or different concentrations of n-3 PUFA-enriched diet (L-PUFA and H-PUFA) for 12 weeks. A) animal weight (n = 10/group). There was no significant difference of the body weights among the three groups. B) Serum ALT level, C) liver histology (upper) and immunohistochemistry (IHC, lower). Kupffer cells were indicated by detection of the F4/80 positive expression of brown yellow staining in cytoplasm (200x magnification), the arrows indicated Kupffer cells. D) Mean (±SD) number of Kupffer cells, ten fields per section from each mouse were analyzed (n = 4/group). (TIF) [file pone.0132741.s001.tif]
